# Supplementary material for: Controlled release of growth factors using synthetic glycosaminoglycans in a modular macroporous scaffold for tissue regeneration
Source: Commun Biol. 2022 Dec 8;5:1349. doi: 10.1038/s42003-022-04305-9 (PMC9732287; doi:10.1038/s42003-022-04305-9)
Supplement: Supplementary file 2 — Supplementary figures [file 42003_2022_4305_MOESM2_ESM.pdf]

Supplementary figures for “Controlled release of growth factors using synthetic glycosaminoglycans in a modular macroporous scaffold for tissue regeneration “

Supplementary figure 1

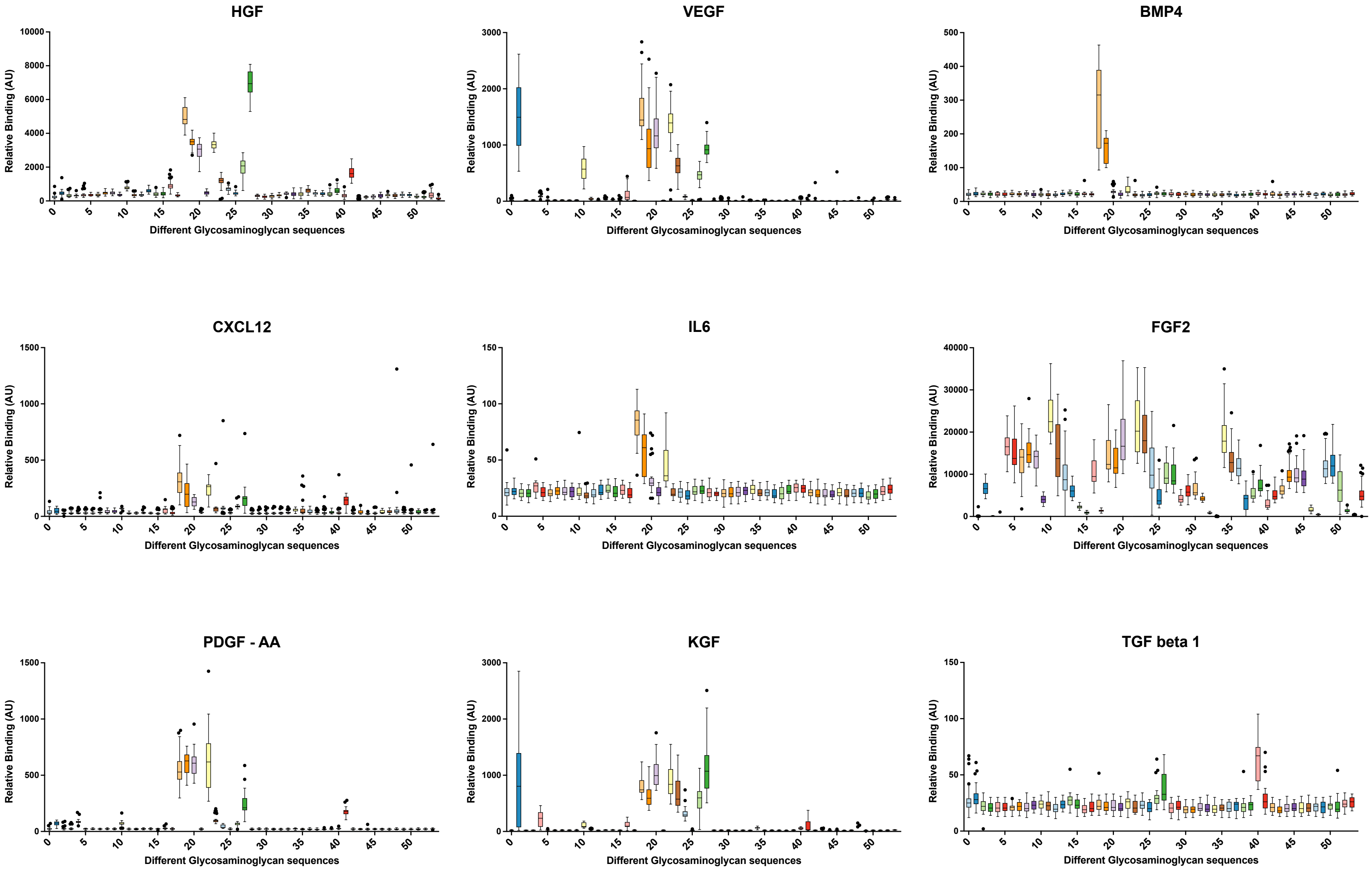

Supplementary figure 1 Relative binding of HGF,VEGF,BMP4,CXCL12,IL6,FGF2,PDGF-AA,KGF and TGF beta 1 in the microarray to show the difference in binding pattern depending on the glycosaminoglycan. The data is shown in a box and whisker (as Tukey) of 36 binding spots for each synthetic glycosaminoglycans

Supplementary figure 2

A) Without GAGs

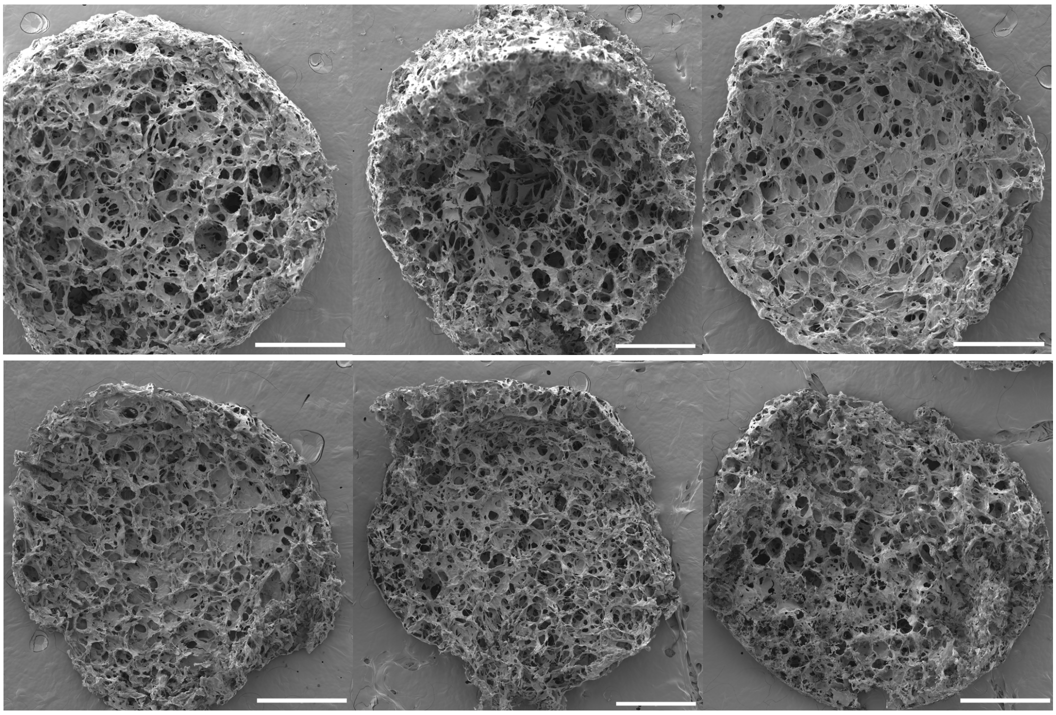

B) With GAGs

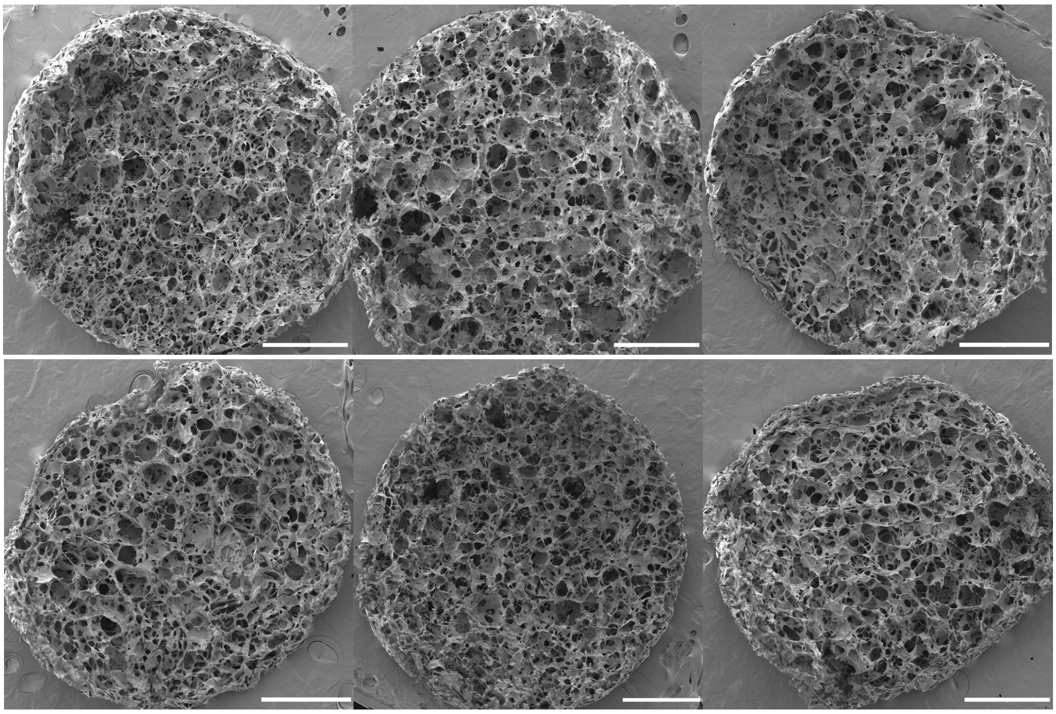

Supplementary figure 2 SEM pictures of cryogels made without GAGs in comparison to those made with GAGs, scale bar is 1000 µm

Supplementary figure 3

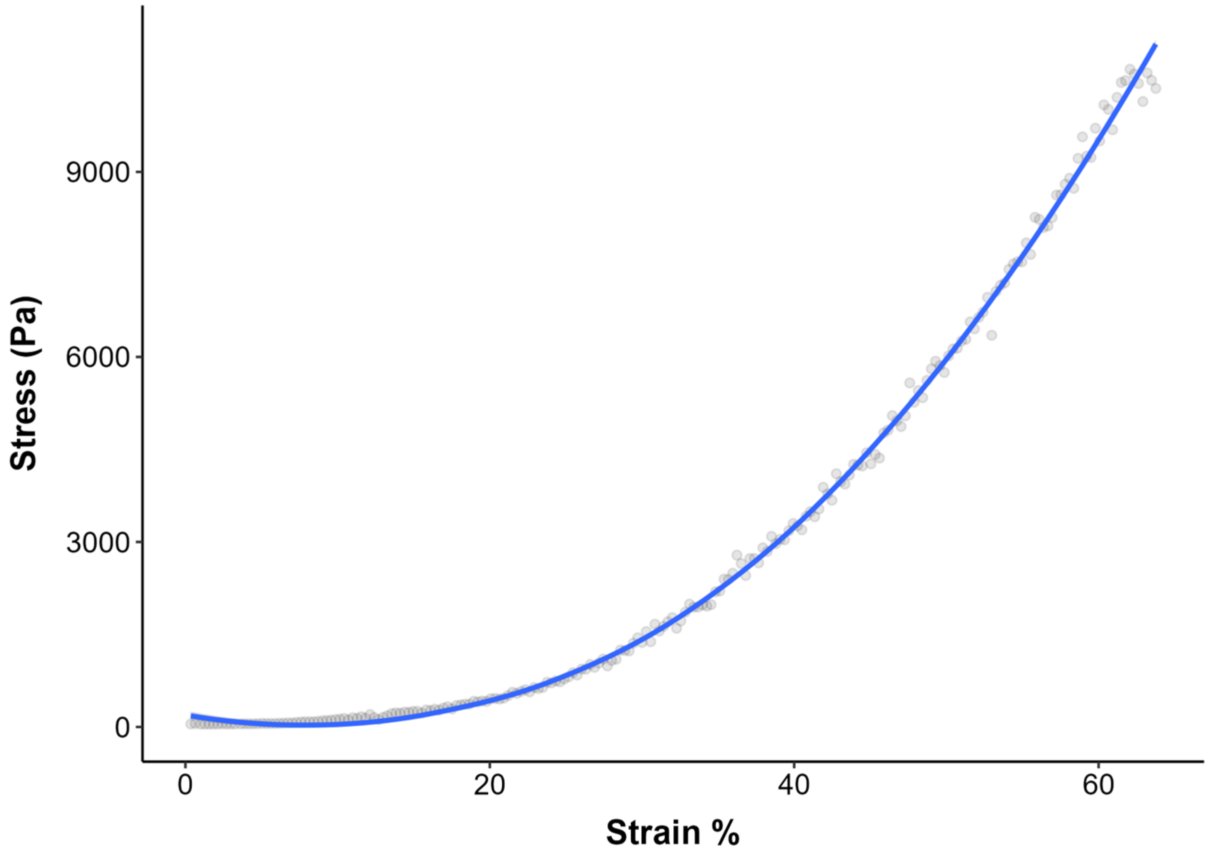

Supplementary figure 3 Stress-strain curve of the ELR hydrogels measured by uniaxial compression tests. The tangential E was calculated considering the adjustment for a linear regression in the first slope of the curve (0-30% strain; n = 3).

Supplementary figure 4

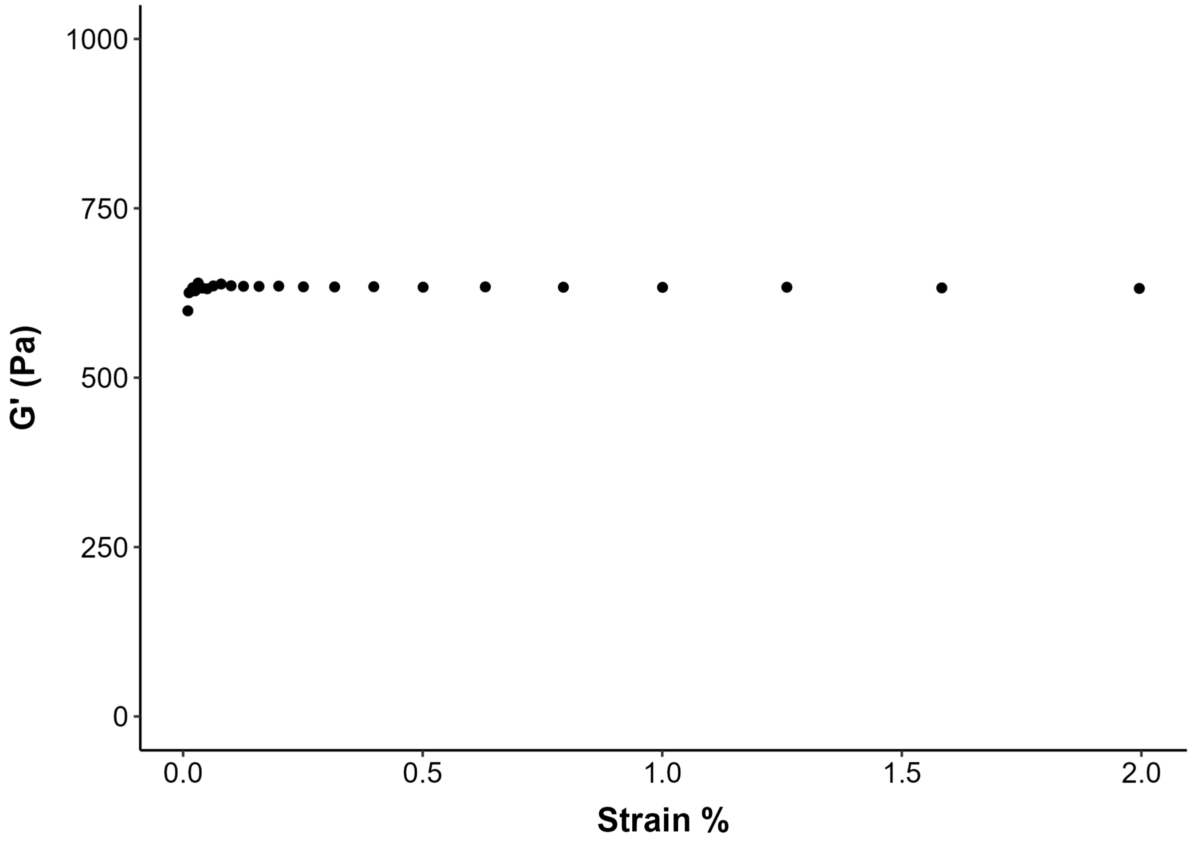

Supplementary figure 4 Strain sweep curve from the oscillatory rheology experiments showing storage modulus (G') versus percentage of strain (from 0 to 2%) for the ELR hydrogels. The result given in the manuscript was determined from the G' value at 1% strain (viscoelastic region; n = 3).

Supplementary figure 5

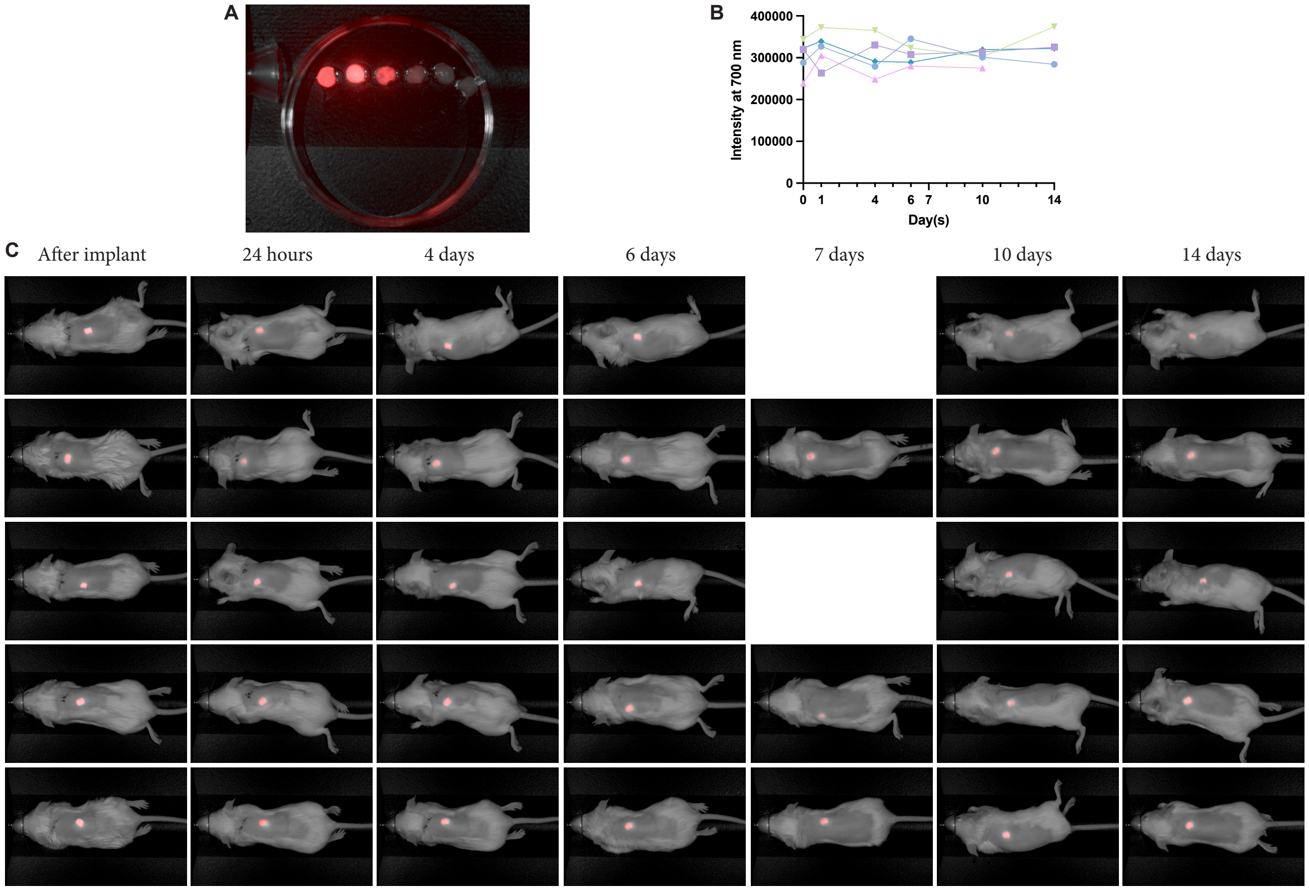

Supplementary figure 5 A) Shows the fluorochrome 680RD-DBCO added in different amount to the cryogel. From left to right, 10^1,10^2, 10^3,10^4,10^5 and 10^6 of diluted fluorochrome was used. For subsequent experiment (B and C) the 100 times dilutions were used as this gave the highest results. In B) and C) cryogels were implanted and followed for 14 days. In B) we can see that there is no loss of signal during the 14 days, due to technical difficulties one of the mice did not give measurable data on the 14 days. C) shows the mice during the experiment, each row represents the same mice with photos taken at each time point. To be sure that the acquiring of the photos did not bleach the samples, two mice were not photographed at day 7 but no difference in signal intensity could be seen.

Supplementary figure 6

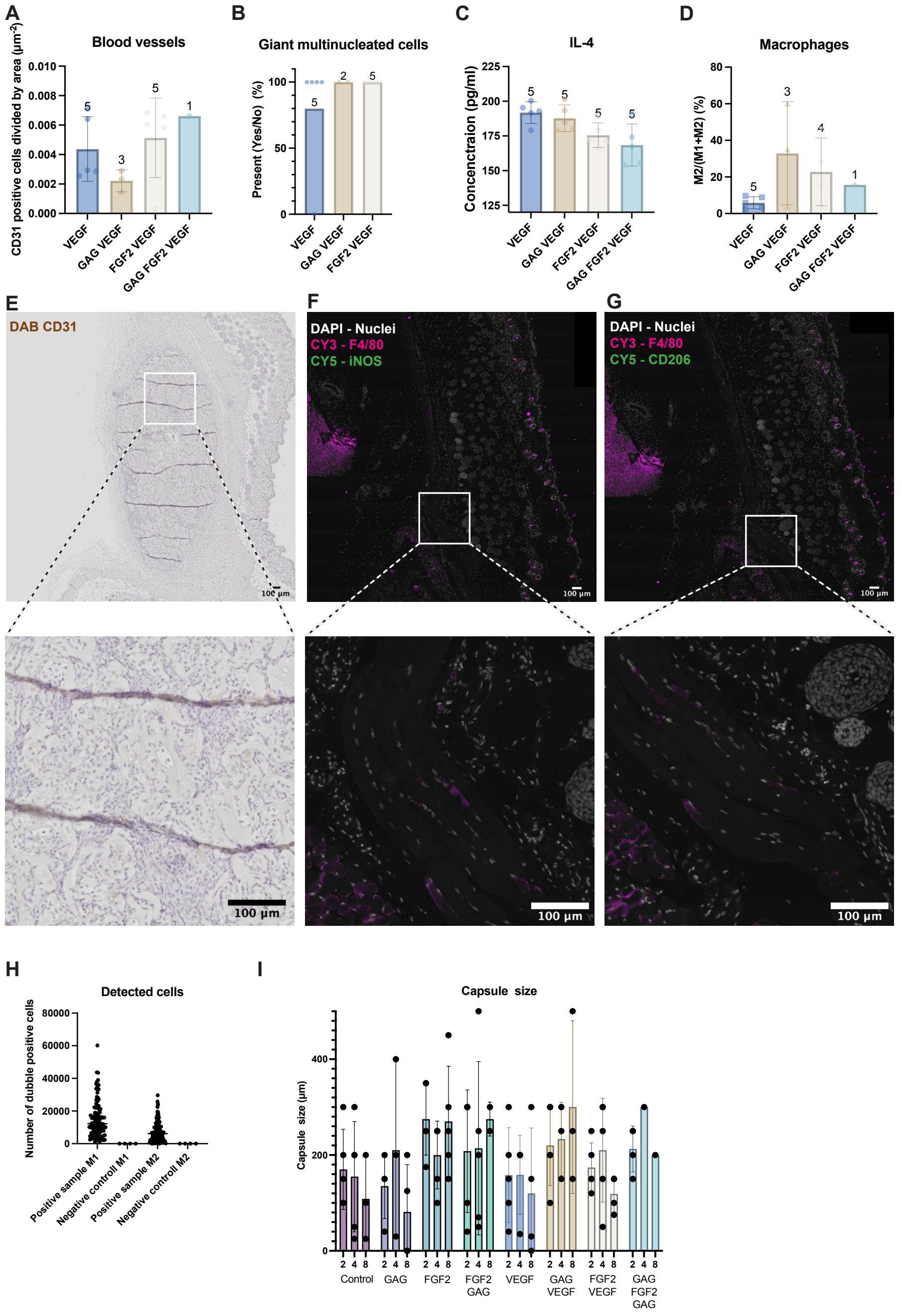

Supplementary figure 6 Supplement figure showing the data from VEGF from the in vivo experiment as well as the negative controls A) The graph shows the number of positive DAB CD31 cells divided by the area showing the highest number of blood vessels in the synthetic glycosaminoglycan and VEGF group. There was a limited number of slides that could be recovered from each sample and therefore all groups do not have n = 5. B) Hematoxylin and Eosin-stained samples were scored for the presence (1) or not presence (0) of multinucleated cells. There was a limited number of slides that could be recovered from each sample and therefore the group GAG FGF2 and VEGF is missing C) Chemokines and growth factor levels were measured in plasma for IL-6, IL-4, VEGF, FGF2, MMP8 and TIMP-1, where a difference only could be observed for IL-4. D) The M2 to total number of macrophages by staining one slide for M1 and one for M2 macrophages, which were then counted and normalized to total cell number dividing M2 with M1+M2. E) The negative control of the DAB staining without primary Ab F and G) The negative control of the M1 and M2 staining without primary Ab, but with DAPI. There is autofluorescence of hair in Alexa 555 (yellow) and from blood clots, blood clots were excluded in the analysis H) Number of double positive cells detected in samples compared to negative controls, there was no detected double positive cell in the negative controls. I) Shows the capsule size in µm for each sample

Supplementary figure 7

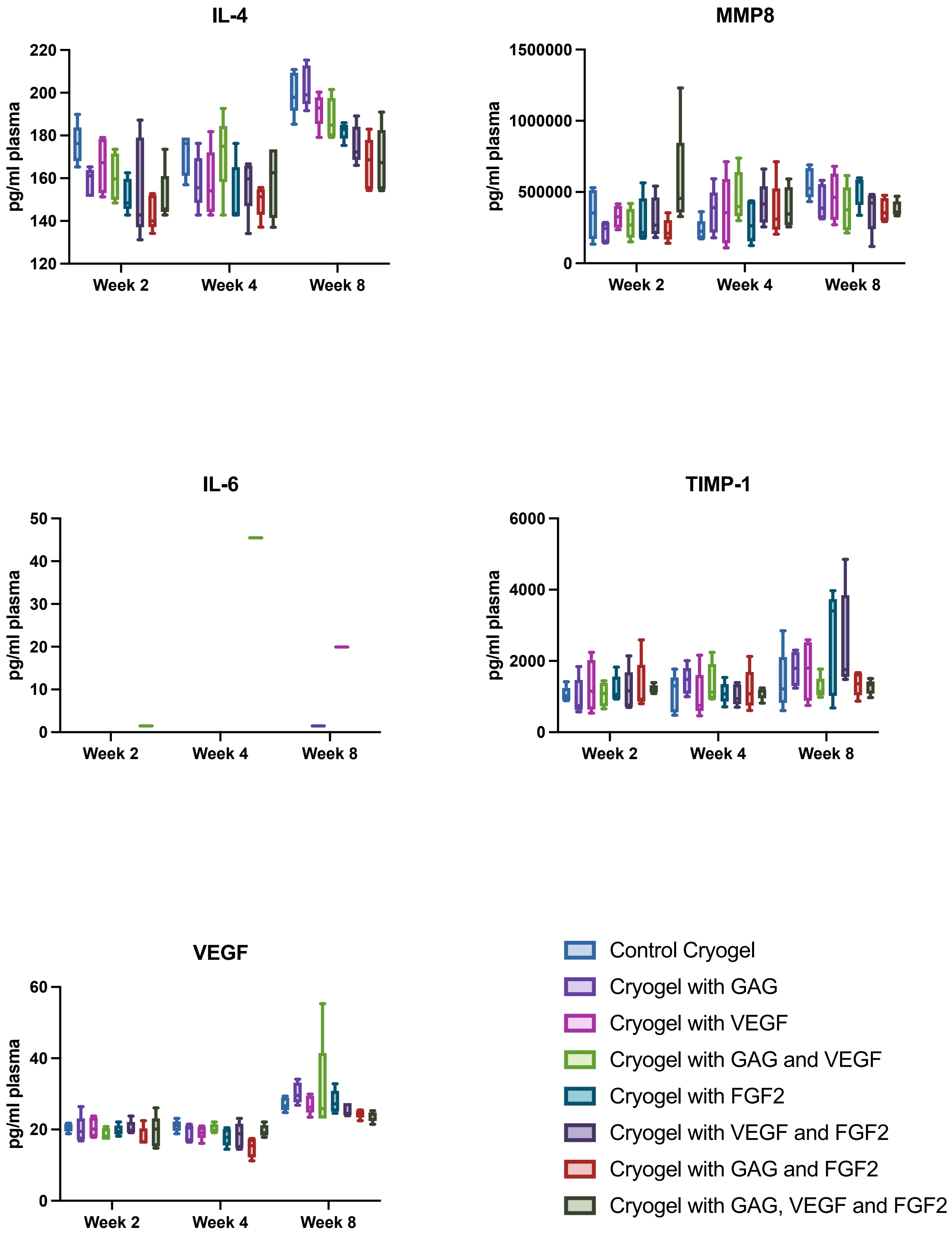

Supplement figure 7 Plotted values from the Luminex assay from the in vivo experiment. The graphs are plotted as box and whiskers with Tukey. There are 5 biological replicates from each group and time point. The raw numbers can be found in Supplement table 3

Supplementary figure 8

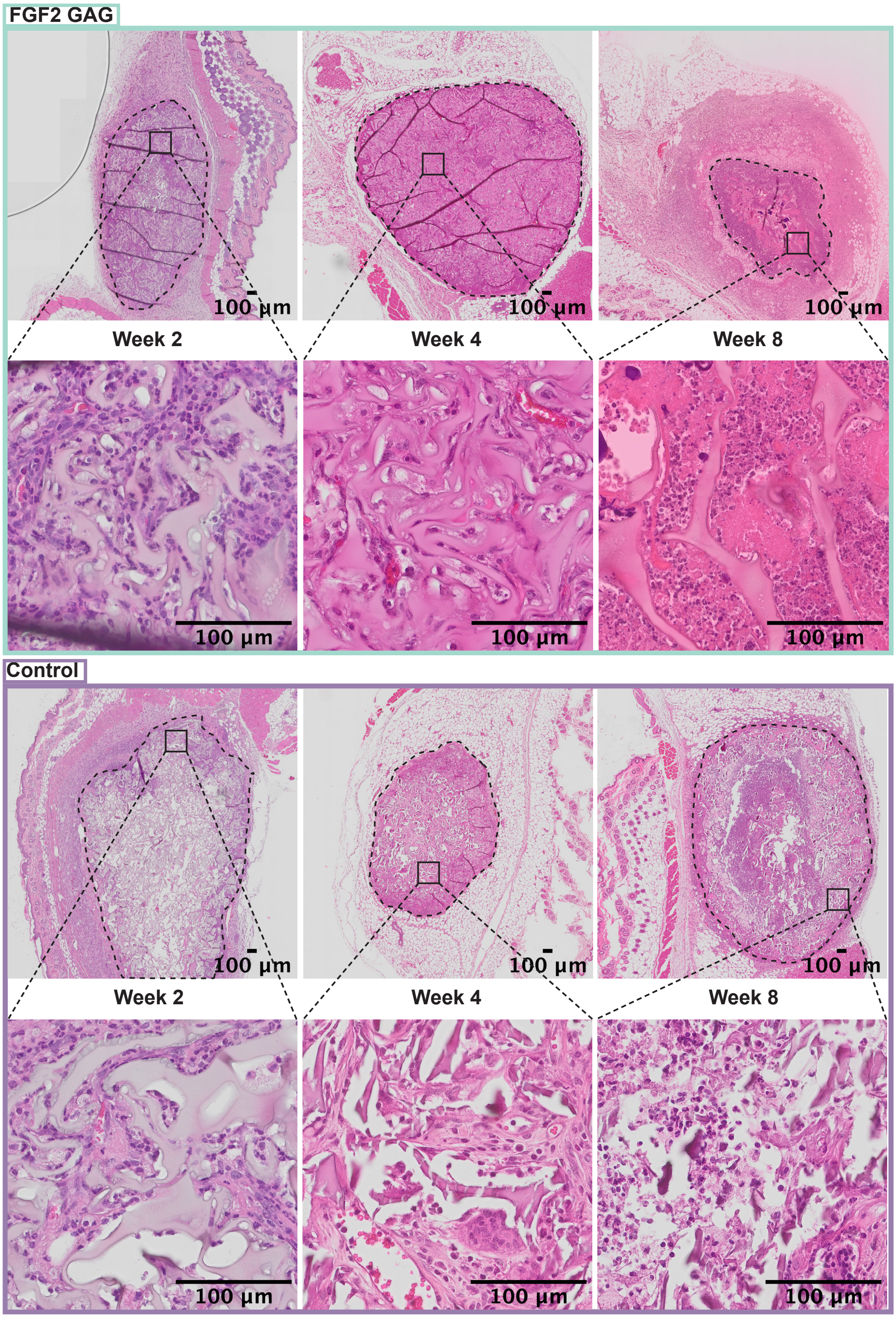

Supplementary figure 8 Hematoxylin and Eosin staining from the in vivo study at the timepoints 2 4 and 8 weeks. In green outline the FGF2 and GAG group can be seen and in purple outline the control elastin like recombinamer can be seen

Supplementary figure 9

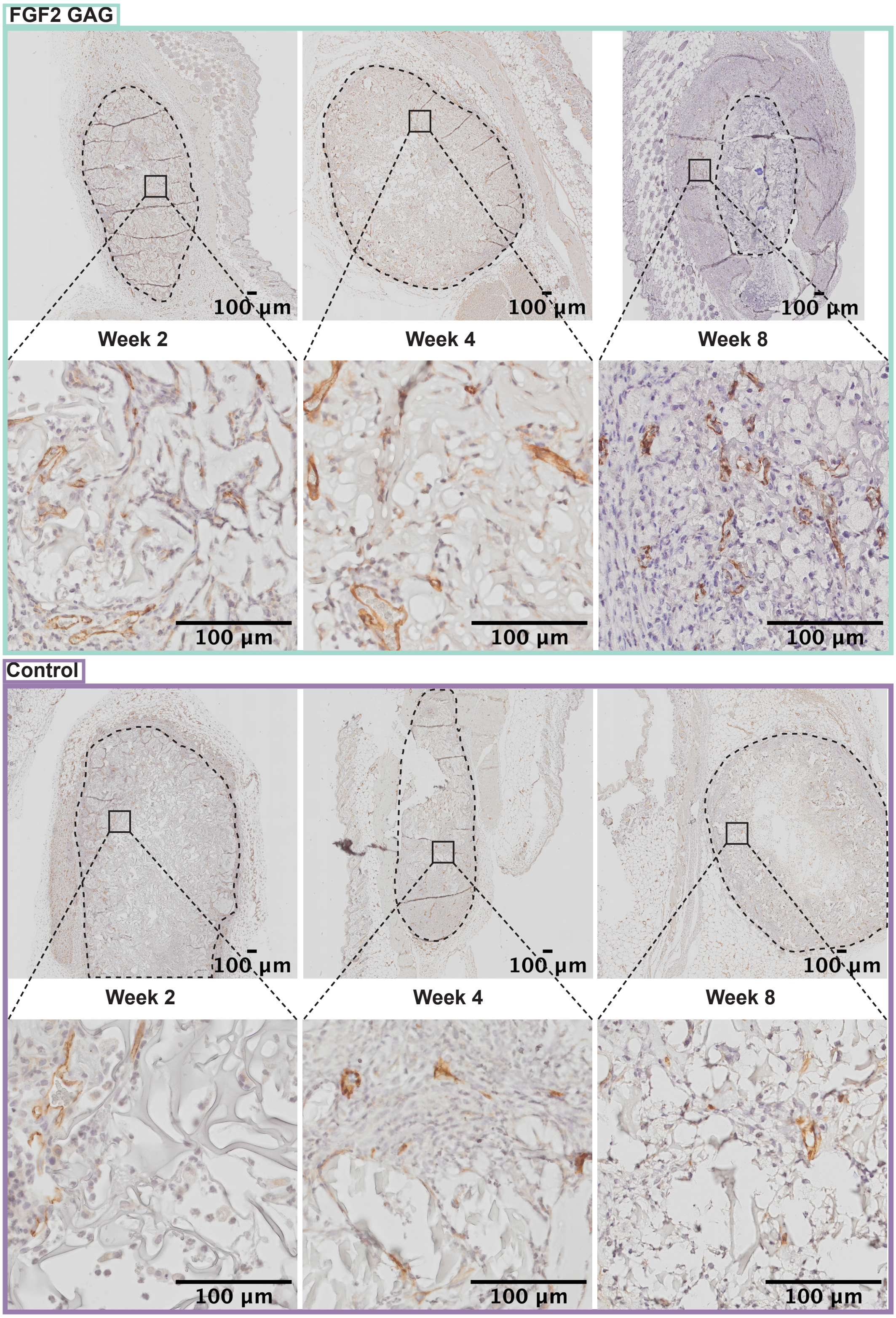

Supplementary figure 9 CD31 DAB staining from the in vivo study at the timepoints 2 4 and 8 weeks. In green outline the FGF2 and GAG group can be seen and in purple outline the control elastin like recombinamer can be seen

Supplementary figure 10

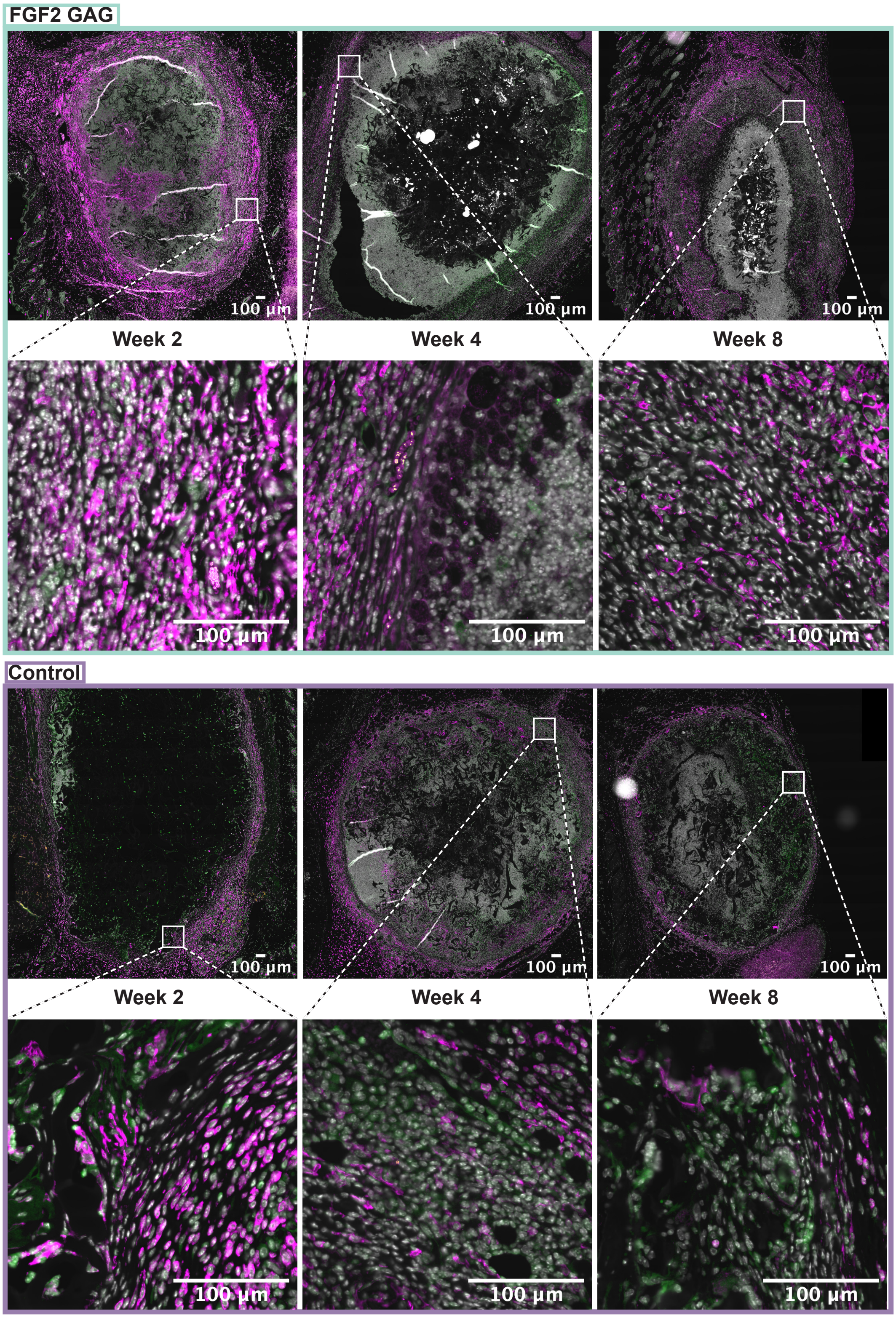

Supplementary figure 10 M1 Macrophage staining from the in vivo study at the timepoints 2 4 and 8 weeks. In green outline the FGF2 and GAG group can be seen and in purple outline the control elastin like recombinamer can be seen. White is DAPI, Magenta is F4/80 and Green is iNOS

Supplementary figure 11

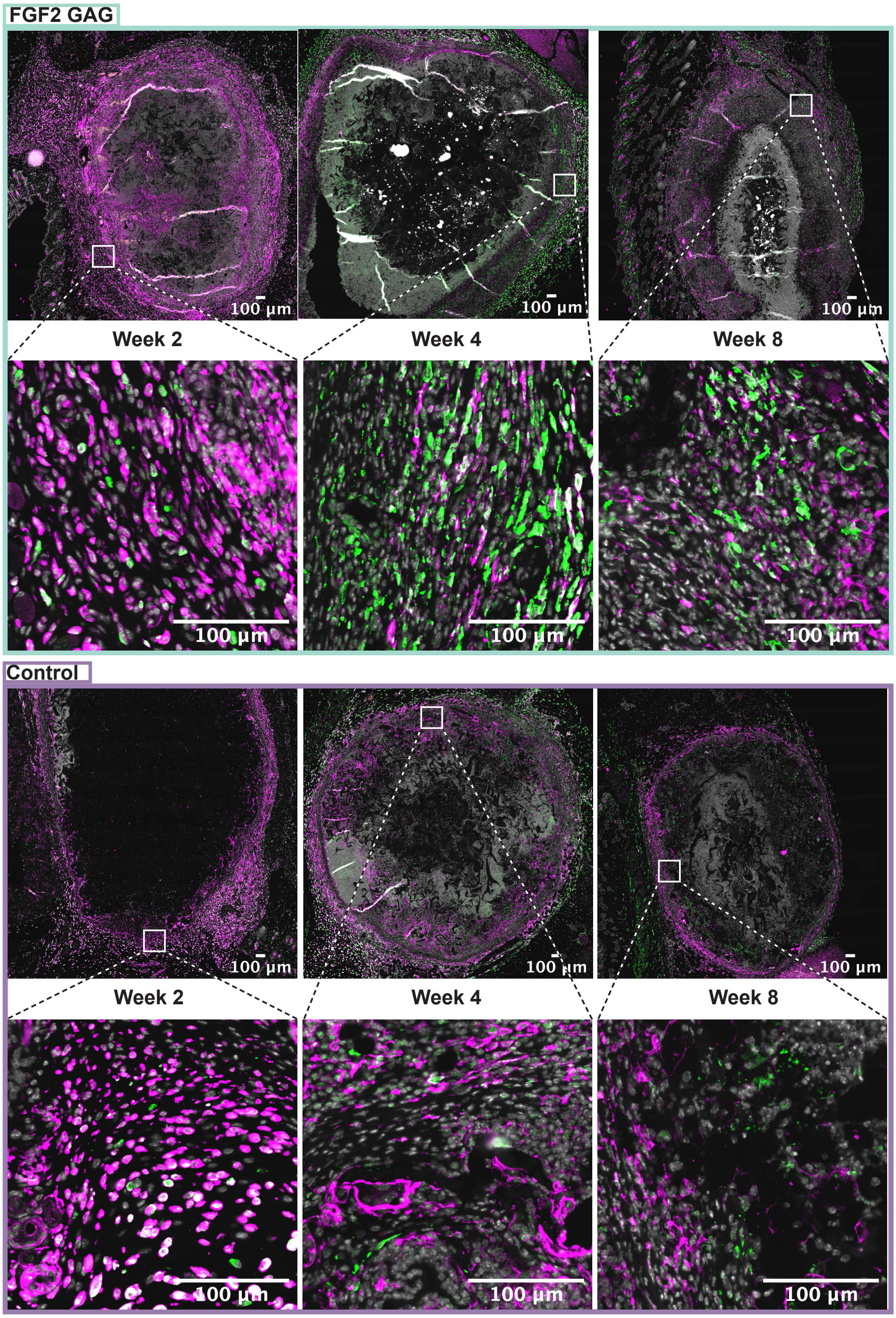

Supplementary figure 11 M2 Macrophage staining from the in vivo study at the timepoints 2 4 and 8 weeks. In green outline the FGF2 and GAG group can be seen and in purple outline the control elastin like recombinamer can be seen. White is DAPI, Magenta is F4/80 and Green is CD206

Supplementary figure 12

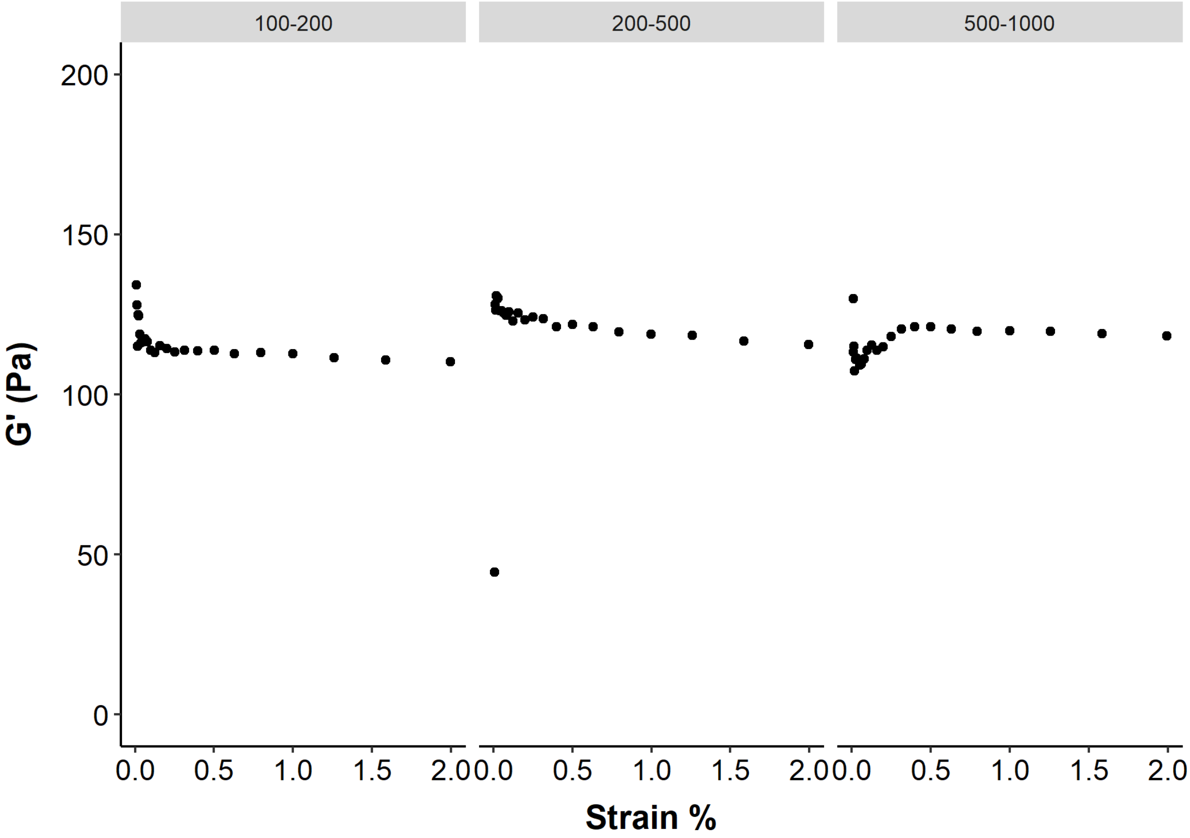

Supplementary figure 12 Strain sweep curve from the oscillatory rheology experiments showing storage modulus (G') versus percentage of strain (from 0 to 2%) for the ELR cryogels with different pore size (n = 3).
