# Supplementary material for: Controlled release of growth factors using synthetic glycosaminoglycans in a modular macroporous scaffold for tissue regeneration
Source: Commun Biol. 2022 Dec 8;5:1349. doi: 10.1038/s42003-022-04305-9 (PMC9732287; doi:10.1038/s42003-022-04305-9)
Supplement: Supplementary file 3 — Description of Additional Supplementary Files [file 42003_2022_4305_MOESM3_ESM.pdf]

Description of Additional Supplementary Files

File name: Supplementary Data 1
Description: The names for the glycosaminoglycans on the microarray.

File name: Supplementary Data 2
Description: The VEGF data from the microarray screening.

File name: Supplementary Data 3
Description: The HGF data from the microarray screening.

File name: Supplementary Data 4
Description: The BMP4 data from the microarray screening.

File name: Supplementary Data 5
Description: The CXCL12 data from the microarray screening.

File name: Supplementary Data 6
Description: The IL6 data from the microarray screening.

File name: Supplementary Data 7
Description: The FGF2 data from the microarray screening.

File name: Supplementary Data 8
Description: The PDGF-AA data from the microarray screening.

File name: Supplementary Data 9
Description: The KGF data from the microarray screening.

File name: Supplementary Data 10
Description: The TGF beta 1 data from the microarray screening.

File name: Supplementary Data 11
Description: The SPR data.

File name: Supplementary Data 12
Description: The data collected for the in vivo study.

File name: Supplementary Data 13
Description: Compression data for Cryogels.

File name: Supplementary Data 14
Description: Compression test for Hydrogels.

File name: Supplementary Data 15
Description: Rehology data for Cryogels.

File name: Supplementary Data 16
Description: Rheology data for Hydrogels.
