# Supplementary material for: Controlled release of growth factors using synthetic glycosaminoglycans in a modular macroporous scaffold for tissue regeneration
Source: Commun Biol. 2022 Dec 8;5:1349. doi: 10.1038/s42003-022-04305-9 (PMC9732287; doi:10.1038/s42003-022-04305-9)
Supplement: Supplementary file 20 — Reporting summary [file 42003_2022_4305_MOESM20_ESM.pdf]

nature portfolio

Corresponding author(s): Zackarias Söderlund

Last updated by author(s): Nov 18, 2022

Reporting Summary

Nature Portfolio wishes to improve the reproducibility of the work that we publish. This form provides structure for consistency and transparency in reporting. For further information on Nature Portfolio policies, see our Editorial Policies and the Editorial Policy Checklist.

Statistics

For all statistical analyses, confirm that the following items are present in the figure legend, table legend, main text, or Methods section.

n/a	Confirmed	
☐	☒	The exact sample size (*n*) for each experimental group/condition, given as a discrete number and unit of measurement
☐	☒	A statement on whether measurements were taken from distinct samples or whether the same sample was measured repeatedly
☒	☐	The statistical test(s) used AND whether they are one- or two-sided<br>*Only common tests should be described solely by name; describe more complex techniques in the Methods section.*
☒	☐	A description of all covariates tested
☒	☐	A description of any assumptions or corrections, such as tests of normality and adjustment for multiple comparisons
☐	☒	A full description of the statistical parameters including central tendency (e.g. means) or other basic estimates (e.g. regression coefficient) AND variation (e.g. standard deviation) or associated estimates of uncertainty (e.g. confidence intervals)
☒	☐	For null hypothesis testing, the test statistic (e.g. *F*, *t*, *r*) with confidence intervals, effect sizes, degrees of freedom and *P* value noted<br>*Give P values as exact values whenever suitable.*
☒	☐	For Bayesian analysis, information on the choice of priors and Markov chain Monte Carlo settings
☒	☐	For hierarchical and complex designs, identification of the appropriate level for tests and full reporting of outcomes
☒	☐	Estimates of effect sizes (e.g. Cohen's *d*, Pearson's *r*), indicating how they were calculated

Our web collection on statistics for biologists contains articles on many of the points above.

Software and code

Policy information about availability of computer code

Data collection	Sierra Analyzer 3.1.36 (Bruker)<br>Mapix 5.5.0 (Innopsys)<br>xPONENT 4.2.1705.0 (Luminex MAGPIX)<br>VS120 (Olympus Corporation)<br>AR2000ex (TA Instruments)<br>ElectroForce 5500 (TA Instruments)<br>Jeol JSM-7800F FEG-SEM<br>Dionex ultimate 3000 (Thermo Fisher)
Data analysis	Sierra Analyzer 3.1.36 (Bruker)<br>Mapix 5.5.0 (Innopsys)<br>VIS 20190808 (Visiopharm)<br>QuPath 0.2.3<br>xPONENT 4.2.1705.0 (Luminex MAGPIX)<br>GraphPad Prism 9 (Graphpad Software)

For manuscripts utilizing custom algorithms or software that are central to the research but not yet described in published literature, software must be made available to editors and reviewers. We strongly encourage code deposition in a community repository (e.g. GitHub). See the Nature Portfolio guidelines for submitting code & software for further information.

Data

Policy information about availability of data

All manuscripts must include a data availability statement. This statement should provide the following information, where applicable:

- Accession codes, unique identifiers, or web links for publicly available datasets
- A description of any restrictions on data availability
- For clinical datasets or third party data, please ensure that the statement adheres to our policy

The datasets generated during and/or analysed during the current study are available from the corresponding author on reasonable request.

Field-specific reporting

Please select the one below that is the best fit for your research. If you are not sure, read the appropriate sections before making your selection.

☒ Life sciences ☐ Behavioural & social sciences ☐ Ecological, evolutionary & environmental sciences

For a reference copy of the document with all sections, see nature.com/documents/nr-reporting-summary-flat.pdf

Life sciences study design

All studies must disclose on these points even when the disclosure is negative.

Sample size	No statistical method was used to determine sample size. Size was based on experience with previous studies.
Data exclusions	Slides from the in vivo studies where no inplant could be found was exluded.
Replication	In vitro experiments were successfully replicated, in vivo experiments were not replicated.
Randomization	Samples were randomly allocated into experimental groups
Blinding	The investigators were not blinded during analysis

Reporting for specific materials, systems and methods

We require information from authors about some types of materials, experimental systems and methods used in many studies. Here, indicate whether each material, system or method listed is relevant to your study. If you are not sure if a list item applies to your research, read the appropriate section before selecting a response.

Materials & experimental systems

n/a	Involved in the study	
☐	☒	Antibodies
☒	☐	Eukaryotic cell lines
☒	☐	Palaeontology and archaeology
☐	☒	Animals and other organisms
☒	☐	Human research participants
☒	☐	Clinical data
☒	☐	Dual use research of concern

Methods

n/a	Involved in the study	
☒	☐	ChIP-seq
☒	☐	Flow cytometry
☒	☐	MRI-based neuroimaging

Antibodies

Antibodies used

(HGF Ab, 0.4 µg/ml, Mouse anti Human, RnD systems, MAB694)
(VEGF Ab, 0.4 µg/ml, Mouse anti Human, RnD Systems MAB293)
(BMP4 Ab, MAB7571, 0.4 µg/ml, Mouse anti Human, RnD systems)
(CXCL12 Ab, MAB350, 0.4µg/ml, Mouse ant Human, RnD Systems)
(IL-6 Ab, MAB2063, 0.4 µg/ml, Mouse ant Human, RnD systems)
(FGF2 Ab, ab92337, 0.5 µg/ml, Rabbit anti Human, Abcam)
(PDGF-AA Ab, MAB221, 0.4 µg/ml , Mouse anti Human, RnD systems)
(KGF Ab, MAB251, 0.4 µg/ml , Mouse anti Human, RnD systems)
(TGF beta 1 Ab, MAB240, 0.4 µg/ml, Mouse anti Human, RnD systems)
(Alexa fluor Plus 647, IgG Alexa 647 plus, Dilution: 1/1000, Goat anti Mouse, Thermo Fisher, A32728,)
(Rabbit a-CD31, Dilution:1/50, Abcam ab28364)
(BrightVision rabbit/HRP , Dilution: No Dilution (Ready to Use), Immunologic, DPVR110HRP)

	(iNOS, Dilution: 1/100, Rabbit anti Mouse, Abcam ab3523)<br>(F4/80, Dilution: 1/25, Rat anti Mouse, Abcam ab16911)<br>(CD206, Dilution: 1/200, Rabbit anti Mouse, Abcam, ab64693)<br>(IgG Alexa 647 plus, Dilution: 1/500, Goat anti Rabbit , Thermo Fisher, A32733)<br>(IgG Alexa 555 plus, Dilution: 1/500, Goat anti Rat, Thermo Fisher, A48263)
Validation	Antibodies were validated by manufacturer

Animals and other organisms

Policy information about studies involving animals; ARRIVE guidelines recommended for reporting animal research

Laboratory animals	Mice, BALB/cJRj, Female, 8 weeks at arrival with minimum 5 days of acclimatization.
Wild animals	This study did not involve wild animals
Field-collected samples	This study did not involve field-collected samples
Ethics oversight	Malmö and Lunds animal experiment ethics committee

Note that full information on the approval of the study protocol must also be provided in the manuscript.
